# Circular RNAs as promising biomarkers in cancer: detection, function, and beyond

**DOI:** 10.1186/s13073-019-0629-7

**Published:** 2019-03-20

**Authors:** Shengli Li, Leng Han

**Affiliations:** 1 Department of Biochemistry and Molecular Biology, McGovern Medical School at The University of Texas Health Science Center at Houston, Houston, TX 77030 USA; 2 Center for Precision Health, The University of Texas Health Science Center at Houston, Houston, TX 77030 USA

## Abstract

Circular RNAs (circRNAs) are 3′–5′ covalently closed RNA rings produced from back-splicing of precursor mRNA in eukaryotes. Recent studies, using both computational and experimental approaches, have allowed advanced characterization of circRNAs, leading the research field into a new era and shedding light on the contribution of circRNAs to disease.

## Circularization diversifies the transcriptome

Circular RNAs (circRNAs) arise through ‘out-of-order’ splicing, which involves covalent ligation between the downstream 5′ splice sites and the upstream 3′ splice sites of precursor messenger RNA (pre-mRNA; a process called back-splicing) [1]. In effect, the generation of circRNAs diversifies the eukaryotic transcriptome, increasing the functional capacity of a gene. circRNAs are generally expressed at lower abundance levels than linear transcripts, and until recent years, they were viewed as splicing noise or ligation artifacts. They can be circularized from many genomic sources, including exons (ecircRNA), introns (ciRNA), exon-introns (EIciRNA), or fusion transcripts (f-circRNA) of parental genes. circRNA expression is widespread among eukaryotic organisms, but importantly exhibits cell-specific and tissue-specific patterns.

Specific factors are involved in the regulation of circRNA biogenesis [1], a process that involves the spliceosomal splicing mechanism wherein RNA-binding proteins participate in the formation of circRNAs by binding to and probably stabilizing the back-splicing process. The splicing factor Quaking (QKI) has been demonstrated to regulate a variety of circRNAs that are involved in the epithelial–mesenchymal transition (EMT) in humans, exemplifying a role for circRNA regulation in a biological process that could have implications for diseases such as cancer [1]. Further investigations of factors that directly or indirectly impact back-splicing are needed to enrich our understanding of the biogenesis of circRNAs.

circRNAs have been shown to function through sponging microRNAs, by interacting with proteins, by regulating the transcription of parental genes, or by encoding polypeptides [1]. For example, Cerebellar degeneration-related protein 1 antisense RNA (CDR1as) participates in the regulation of genes that contain miR-7-binding sites through competitive interaction with miR-7, whereas circFOXO3 acts as an accelerator of cardiac senescence by interacting with the transcription factor E2F1 and the anti-senescent protein ID-1. circRNAs can also regulate the transcription of their parental genes specifically. For example, circEIF3J has been shown to enhance the expression of its parental gene by interacting with U1 small nuclear ribonucleoproteins and with Pol II. CircZNF609 is an example of a circRNA that can be translated into a polypeptide, which may play a functional role in regulating myoblast proliferation [1]. Dysregulation of circRNA expression could lead to alterations in these processes, and there is increasing evidence of a role for circRNAs as regulatory RNA molecules in tissue homeostasis and in human diseases such as cancer. Therefore, it is essential that circRNAs are detected and quantified accurately so that their functions can be investigated further.

## Use of RNA deep-sequencing technology to identify circRNAs

The advent of high-throughput RNA deep-sequencing technology (RNA-seq) brought the encouraging discoveries that, rather than being sequencing artifacts, circRNAs are pervasively expressed in human genes [1] and can be validated by quantitative PCR (qPCR) [2]. The choice of RNA library preparations before sequencing will affect the detection of circRNAs [2]. Currently, the most commonly used RNA-seq library preparation strategies for circRNA detection are Ribo-Zero (ribosome RNA (rRNA) depletion) and RNase R. Ribo-Zero libraries include both linear and circular RNAs after rRNA depletion, and thus do not provide tailored enrichment of circRNAs. Their advantage is that they retain ample RNA information to facilitate downstream analysis. By contrast, the RNase R library digests linear RNA while the covalently closed loop structure of circRNAs allows them to elude exonucleolytic degradation, resulting in the enrichment of circRNAs. The digestion of linear RNAs limits the application of RNase R libraries in further downstream analysis.

In a recent study, Vo et al. [3] employed exome capture RNA-seq to detect circRNAs. By targeting gene bodies, they achieved better enrichment for circRNA than that in the Ribo-Zero libraries, while simultaneously preserving linear RNAs. Thus, by achieving a balance between the enrichment of circRNAs and circular-to-linear ratios, their protocol complements conventional Ribo-Zero or RNase R strategies for systematic investigations of circRNAs. This protocol requires less than 5 μg of total RNA, suggesting that it will offer a significant advantage when used for clinical biospecimens that provide limited extracted RNA. Although the strategy is limited to circRNAs in known exonic regions, and thus probably misses circRNAs that originate from intronic and intergenic regions, Vo et al. [3] successfully characterized circRNAs in more than 2000 tissue samples and 28 cell lines. They also identified read-through circRNAs, a novel class of circRNAs involving exons that originate from multiple genes. Furthermore, Vo et al. [3] built a comprehensive catalog of circRNAs in human cancers, MiOncoCirc. This is a much larger compendium than any other circRNA data resource, including the Cancer-Specific CircRNA Database [4]. By exploring MiOncoCirc, Vo et al. [3] were able to show a strong tissue-specific pattern of circRNAs across different cancer types. They also demonstrated that circRNAs that were identified in prostate cancer tissue samples could be reliably detected in urine samples, suggesting the exciting possibility that circRNAs could have potential for use as biomarkers in the noninvasive diagnosis of human cancers. Thus, MiOncoCirc is a valuable resource that will promote the identification of novel circRNAs as diagnostic and therapeutic targets.

## Computational approaches to detect circRNAs

Alongside the burst of RNA-seq data, a variety of computational algorithms for the identification and visualization of circRNAs have recently been developed [5]*.* Most tools are based on detecting back-splicing junctions (BSJs), which are junctions between sequences that occur in the order opposite to that in the reference genome, indicating circularity [6]. These approaches can be classified as split-alignment-based approaches (i.e., reads spanning BSJs are split into segments and then aligned to a reference sequence using tools such as CIRCexplorer, CIRI, and find_circ) or pseudo-reference-based approaches (i.e., in which a pseudo-reference based on all possible BSJs is constructed and the reads are aligned against this pseudo-reference using tools such as KNIFE, NCLscan, and PTESFinder) [6]. Diverse circRNA transcripts might be formed from a single parental gene, however, and to date these algorithms have limited power to detect and quantify the internal structures of circRNAs accurately using the same BSJs. By considering the internal components of circRNA, Zheng et al. [7] proposed a new strategy, reverse overlap (RO), to reconstruct full-length circRNAs*.* The CIRI-full algorithm combines both RO and BSJ reads to allow quantification of circRNAs at the isoform level and is better than existing methods at detecting low-abundance circRNAs. Using this powerful algorithm, Zheng et al. [7] were able to probe for links between disease and isoform specificity; for example, they observed an isoform switch in circZDBF2 from a 447-nucleotide (nt) isoform in normal liver tissues to a 334-nt isoform in liver cancer, providing a candidate for future functional and/or biomarker analysis. CIRI-full promotes the accurate quantification, differential analysis, and alternative splicing analysis of circRNA transcripts and will greatly improve our understanding of circRNA at up to isoform-level resolution.

## Functional characterization of circRNAs

Current analyses indicate that perturbation of circRNAs is widespread in human cancers [1]*.* One of the best-known circular RNAs, CDR1as, has been shown to promote (by acting as a sponge for miR-7) the upregulation of oncogenic factors (such as CCNE1 and PIK3CD) that are targeted by miR-7, thus regulating the proliferation of tumor cells [1]. Targeting these functional circRNAs, for example, by interfering with their biogenesis or their interactions with antisense oligonucleotides, might be a promising therapeutic strategy for cancer [8]. In a recent study, Chen et al. [9] characterized circRNAs in prostate cancer patients and demonstrated that altered circRNAs were associated with prostate cancer progression. Interestingly, they showed that circRNA abundance was significantly associated with more read-through and fusion events, highlighting the potential link between fusion events and circRNA biogenesis. Remarkably, a genome-wide loss-of-function screen using small hairpin RNA to deplete circRNAs specifically revealed a total of 171 circRNAs that were essential for cell proliferation in prostate cancer. These essential circRNAs showed functions that were distinct from those of their linear mRNA counterparts; for example, circular casein kinase 1 gamma 3 (circCSNK1G3) promoted cell growth by interacting with miR-181. This study also implies the contribution of transcriptome diversity in human cancer by revealing the functional pathological significance of circRNAs [9].

## Future directions and therapeutic potential

Despite advancements in the development of treatment options for cancer, most cancer types continue to lack fully characterized and effective targeted therapies. The identification of circRNAs as targets for novel cancer therapies, as well as prognostic and diagnostic tools, represents a promising frontier. In particular, the stable circular structure of circRNAs lengthens their half-life, especially in cell-free samples (such as blood and urine), creating potential for the use of circRNAs as biomarkers in patient samples from noninvasive sources. For example, abundant and stable circRNAs have been detected in human blood exosomes and therefore hold promise in the early diagnosis of cancers [10].

Despite recent advances in characterizing circRNAs in human cancers, significant challenges remain because developing circRNA-targeted therapy will require a deeper understanding of the molecular features, biogenesis, and functional effects of circRNAs in cancer cells. The refined detection methods used in the recent studies described above are leading the way into a new era of understanding the features and functions of circRNAs, providing great opportunities to address the remaining challenges. It is expected that the computational methods and experimental systems established in cancer research will be applicable to other diseases, thereby greatly leveraging the impact of these approaches.

## Abbreviations

BSJ: Back-splicing junction
CDR1as: Cerebellar degeneration-related protein 1 antisense RNA
circRNA: Circular RNA
RNA-seq: High-throughput RNA deep-sequencing technology
